# Incidental Metastatic Meningioma Presenting as a Large Liver Mass

**DOI:** 10.1155/2018/1089394

**Published:** 2018-05-07

**Authors:** Ifeyinwa E. Obiorah, Metin Ozdemirli

**Affiliations:** Department of Pathology, MedStar Georgetown University Hospital, Washington, DC, USA

## Abstract

Meningiomas are slow growing neoplasms of the central nervous system (CNS). Most of the tumors are benign and distant metastasis from a benign meningioma is rare. Metastasis to the liver, although rare, usually presents with hypoglycemia or occurs in conjunction with a clinical history of an intracranial meningioma or following the resection of a prior CNS meningioma, thus making clinical diagnosis relatively easy. Here we present an unusual case of metastatic meningioma to the liver in a 54-year-old female who presented with an incidental liver mass by ultrasound. Her clinical history and physical examination were unremarkable. A partial hepatectomy revealed a meningioma on histology. Further investigation by imaging studies showed a frontal parasagittal dural mass which was confirmed to be a World Health Organization (WHO) grade 1 meningioma. To our knowledge, this is the first report of a clinically silent metastatic meningioma to the liver without either a concurrent or a previous history of meningioma. Precise diagnosis of this challenging case requires high clinical suspicion, histopathology, and immunohistochemistry.

## 1. Introduction

Meningiomas, which are tumors originating from the meninges, are typically benign. Extracranial metastasis is rare and they are sporadically reported in form of case reports and constitute about 0.1% of cases [1]. The major sites usually involved by meningioma include the lungs, axial bones, pleura, mediastinum, and lymph nodes [1, 2]. Metastasis to the liver is rare [3] and are usually symptomatic [4, 5] or may occur simultaneously with a meningioma occurring in the central nervous system (CNS) [6, 7]. Metastatic meningioma to the liver can occur as a recurrence; years after a primary CNS meningioma has been surgically resected [8, 9]. We present a challenging case of metastatic meningioma to the liver that was clinically silent and discovered by imaging during a routine health check. To our knowledge this is the first report of a hepatic metastatic meningioma without clinical symptoms or prior or current history of a CNS meningioma.

## 2. Case Presentation

**Figure 1:**
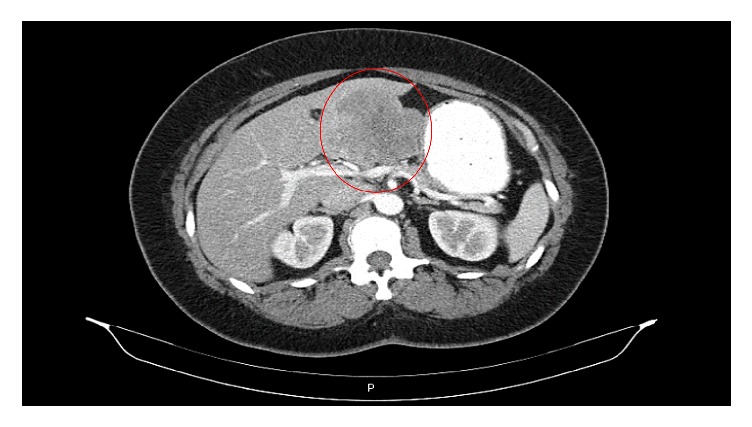
Contrast enhanced computed tomography scan. The image demonstrates a large left hypoattenuating hepatic lobe mass measuring 7.8 × 7.8 cm.

**Figure 2:**
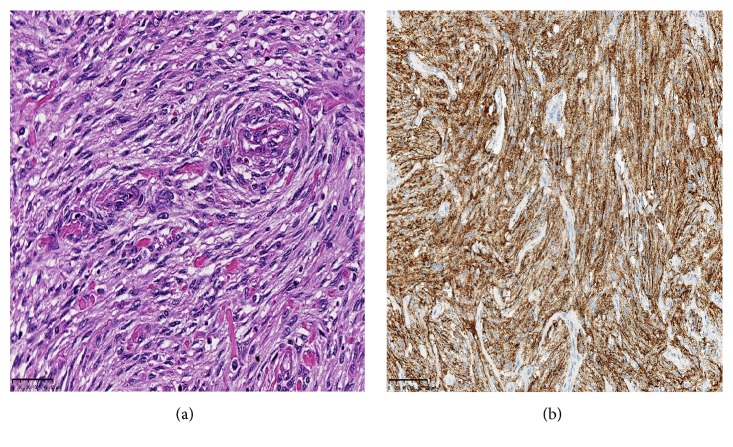
Histologic section of the liver mass. (a) Sections of the liver showing sheets of spindle cells with a whorled pattern in a collagen rich matrix (hematoxylin-eosin, 200x). (b) The neoplastic cells are positive for epithelial membrane antigen by immunohistochemistry (200x).

**Figure 3:**
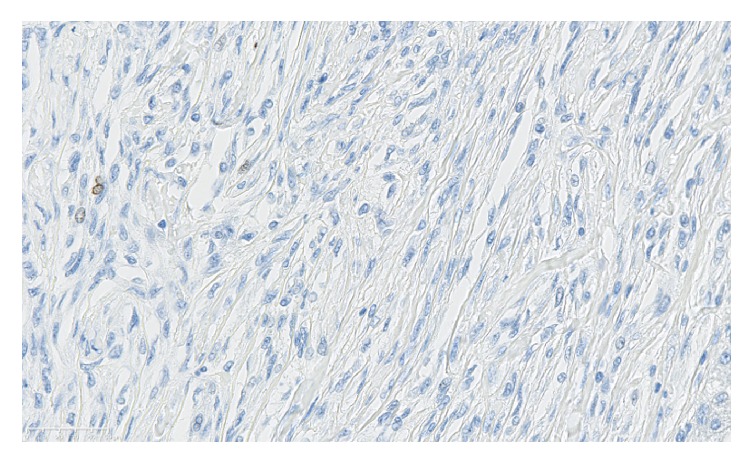
Immunohistochemistry with MIB-1. Approximately 3% of the neoplastic cells stain for MIB-1 by immunohistochemistry (400x).

**Figure 4:**
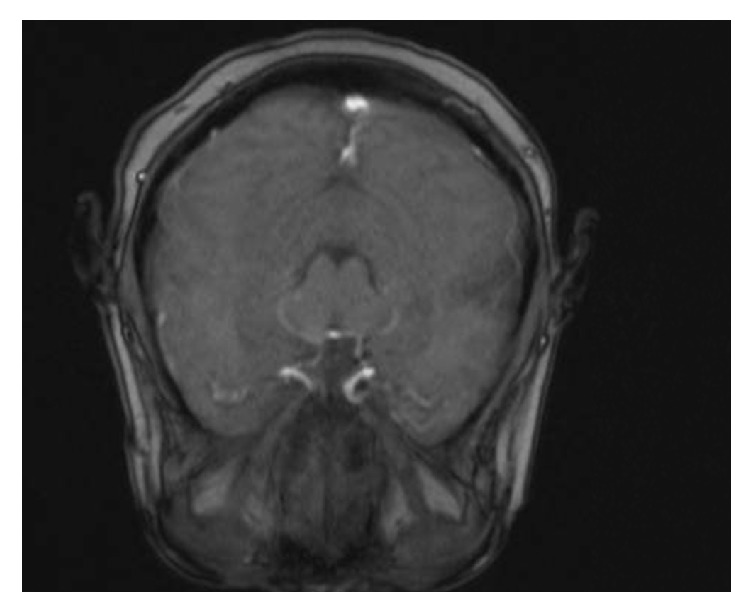
Magnetic resonance imaging of the brain mass. The image shows a small enhancing parasagittal mass.

A 54-year-old woman was referred to our hospital for a recent identification of a left hepatic lobe mass on ultrasound which was performed for mildly elevated liver enzymes during a routine health care check. Her clinical history and physical examination were unremarkable. Liver function results obtained were as follows: aspartate aminotransferase 88 U/L and alanine aminotransferase 99 U/L, alkaline phosphatase 59 U/L, albumin 3.2 g/dl, bilirubin total 1.0 mg/dl, and bilirubin direct 0.2 mg/dl. A contrast enhanced computed tomography scan (CT) showed a hypoattenuating 7.8 cm lobulated mass in the left hepatic lobe (Figure 1). The mass showed decreased enhancement in both the arterial phase and the delayed phase of the contrast CT and the findings were not specific for a particular liver lesion. No other masses were found in the abdomen and pelvis. The differential diagnosis at the time included a hepatocellular neoplasm, cholangiocarcinoma, or a metastatic tumor. Biochemical investigation for viral hepatitis and alpha fetoprotein was negative. A colonoscopy and a chest CT were negative for tumors. She was a nonalcoholic but she had a past history of taking birth control, and it was felt that this could also possibly be a hepatic adenoma. Based on the size of the mass, the patient decided that she wanted the mass to be removed instead of proceeding with a liver biopsy. The patient was scheduled for surgery, during which an intraoperative consultation with a liver biopsy was performed. The biopsy findings were positive for a spindle cell neoplasm with uncertain etiology and a differential diagnosis of a gastrointestinal stromal tumor or a sarcoma. The surgeon proceeded with a partial hepatectomy and the mass was sent to pathology for examination. Histologic sections of the mass showed sheets of spindle cells with mild cytologic atypia, arranged in fascicles and whorls with intervening bands of collagen (Figure 2(a)). A differential diagnosis of meningioma, sarcomatoid carcinoma, melanoma, or a mesenchymal tumor was considered. On immunohistochemistry, the tumor cells were positive for epithelial membrane antigen (EMA) (Figure 2(b)), vimentin, focally positive for progesterone receptor (20%), and negative for cytokeratin, HepPar 1, S-100, CD34, CD117, factor-VIII, CD31, human melanoma black 45 (HMB-45), inhibin, TTF-1, estrogen receptor, and smooth muscle actin. MIB-1 proliferative index was approximately 3% (Figure 3). These results confirmed the diagnosis of metastatic meningioma. To identify the primary source of the meningioma, a magnetic resonance imaging of the CNS was performed, which revealed a 1.4 cm bifrontal parasagittal dural mass (Figure 4). The patient underwent complete resection of the mass at an outside institution and histologic examination of the lesion confirmed the presence of a WHO grade 1 meningioma with bland spindle cells with minimal mitosis and a MIB-1 proliferative index of <3%. Yearly postsurgical imaging of the CNS and the liver did not reveal any residual disease. Six years later, the patient is currently disease-free, without any evidence of recurrence or metastasis.

## 3. Discussion

Meningioma is one of the most common benign tumors of the CNS. However, extracranial metastasis from meningioma is exceedingly rare and the main sites that were reported include the lungs, axial bones, pleura, mediastinum, and lymph nodes [1, 2]. The most important predictive factor for recurrence and metastasis is the grade of the tumor according to the World Health Organization criteria [2]. Other predictive factors include high cellularity, mitotic rate, nuclear atypia, presence of foci of necrosis, and invasion of adjacent structures [9]. However, the presence of these features is not essential for the occurrence of extracranial metastasis. Any histologically benign meningioma has the potential to metastasize [1]. However metastasis from a histologically benign meningioma tends to occur following multiple surgeries for local recurrences [10]. A review by Forest and colleagues [2] reported fifteen cases of WHO grade 1 meningioma occurring in the central nervous system which showed metastasis to various organs; however all cases had a prior diagnosis of meningioma. In our patient, although the primary meningioma in the brain was small and benign (WHO grade 1), it resulted in a large metastatic mass in the liver which was an unusual presentation since the patient did not have any prior surgery or previous occurrence.

Metastasis of meningioma to the liver is uncommon. Although the primary route of dissemination of extracranial meningioma is poorly understood, the proposed methods of spread include the venous system, lymphatics, or the cerebrospinal fluid. The most common reported route of metastasis is via the venous circulation. Possible hypothesis of dissemination of intracranial meningioma to extracranial organs, including the liver, is through the vertebral venous system that connects the veins of the skull, spinal canal, and vertebral column to the thoracoabdominal wall [1, 11] or metastatic dissemination through the jugular veins which transmits tumor cells to the cervical organs, lungs, liver, and other organs [12, 13]. Metastasis can occur through the lymphatic vessels through invasion of the cranial nerves [14]. Finally, intracranial meningioma spreads to other organs through the cerebrospinal fluid in 15% of cases [1]. An unusual mechanism of metastatic spread was reported by Moir and coworkers, who described a hepatic metastasis from an intracranial meningioma through a ventriculoperitoneal shunt [7].

The most frequent presentation of metastatic meningioma to the liver includes hypoglycemic symptoms [4, 5, 15, 16]. Although the cause of hypoglycemia is not known it is thought to be related to the massive hepatic parenchymal replacement with the metastatic neoplasm and the resultant depletion of glycogen stores together with excessive glucose utilization by the tumor tissue [5]. Presence of hypoglycemia in a patient with meningioma should raise the suspicion of metastasis to the liver. Other clinical presentations of hepatic metastatic meningioma include abdominal pain, nausea, vomiting [7], and massive hepatomegaly [17]. Metastatic meningioma to the liver can be detected during work-up of patients with symptomatic primary CNS meningioma [6, 7] or following resection a previous meningioma [8, 9] thus making diagnosis relatively easy. However, a diagnosis of metastatic meningioma in the absence of a clinical history or clinical signs can be very challenging to both the clinician and the pathologist. The key to diagnosis is the histologic identification of spindled cells with a whorling pattern. By immunohistochemistry, meningioma is typically positive for EMA, progesterone receptor, and vimentin and negative for cytokeratin with weak or negative staining for S-100 [18]. A negative stain for cytokeratin excludes a carcinoma. Melanoma was excluded by a negative stain for HMB45 and S100. A mesenchymal tumor will be positive for vimentin or smooth muscle actin, desmin, or CD117.

Treatment of primary CNS meningioma includes complete resection of the tumor by either surgery or radiosurgery and adjuvant radiotherapy if there is subtotal removal of the tumor [1–3]. There is no established treatment for metastatic meningioma, but surgical resection for liver metastasis has shown good prognosis and survival [6, 7]. If asymptomatic, the liver metastasis can be managed by observation using imaging studies [9]. Rampurwala and colleagues reported a case of metastatic meningioma to the liver that was managed by observation for 5 years and the patient remained stable. However, another patient with pulmonary meningioma, managed by watchful waiting per request of the patient died within 70 days of developing hepatic metastasis due to disease progression [19]. Our patient was successfully managed with surgical resection and due to the indolent nature of the tumor; thus surgical resection of the tumor should be considered in resectable tumors. Patients with hypoglycemia have been successfully treated with chemoembolization of the liver mass [4, 5] and may be performed in symptomatic tumors with a large bulky liver mass. Chemotherapy has shown limited or no proven benefit in meningioma. At present the prognostic significance of treatment of metastatic melanoma is difficult to evaluate due to limited data available. Therefore, the ideal therapy for metastatic hepatic meningioma is yet to be determined.

Metastatic meningioma to the liver should be considered in any liver mass with a spindled morphology even in the absence of any clinical symptoms or clinical history of meningioma. Although our case was benign and of a low grade, it confirms that benign meningioma can acquire malignant potential and metastasize to distant organs. Thus, early diagnosis with complete surgical resection is important to improve overall disease survival.
